# Autonomous scheduling mechanism based on energy awareness for improving resource allocation in serverless IoT edge

**DOI:** 10.1038/s41598-025-04214-x

**Published:** 2025-07-07

**Authors:** Mohsen Ghorbian, Mostafa Ghobaei-Arani, Leila Esmaeili

**Affiliations:** Department of Computer Engineering, Qo.C., Islamic Azad University, Qom, Iran

**Keywords:** Autonomous energy-aware scheduler (AEAS), Pre-scheduling, Energy optimization, Adaptive provisioning, Serverless resilience, Failure resistance, Energy grids and networks, Power distribution, Power stations

## Abstract

The energy-aware scheduling mechanism in serverless computing enables systems to be allocated to IoT devices connected to the network’s edge efficiently based on the energy status of active nodes. In this approach, resource allocation is performed in real-time and according to the energy changes of the nodes, the complexity of which arises from the direct dependence of the energy level on the capacity to respond to requests. This choice is complex since allocating the necessary resources to process requests depends on the energy available in the active nodes. Therefore, to optimize resource allocation and increase access time, selecting and executing pre-schedulers is necessary based on the prediction of the energy level of the active nodes. In this article, we introduce a scheduler selection mechanism called an autonomous energy-aware scheduler, whose design is based on the energy position of the active nodes in the network. In addition, a mechanism for improving system uptime is proposed to increase the duration of the energy reduction of active nodes. The efficiency of the proposed approach was evaluated utilizing three separate load distribution patterns (exponential, Poisson, and exponential-Poisson), and the results indicate the prevention of energy waste and an average reduction of 1.66% in energy consumption. Also, the network uptime is improved by 8.6% compared to other methods. In addition, the proposed method has maintained performance continuity while ensuring failure resistance in all situations. The results demonstrate the high efficiency of the proposed approach in optimizing energy consumption and enhancing the resilience and stability of serverless systems.

## Introduction

**Fig. 1:**
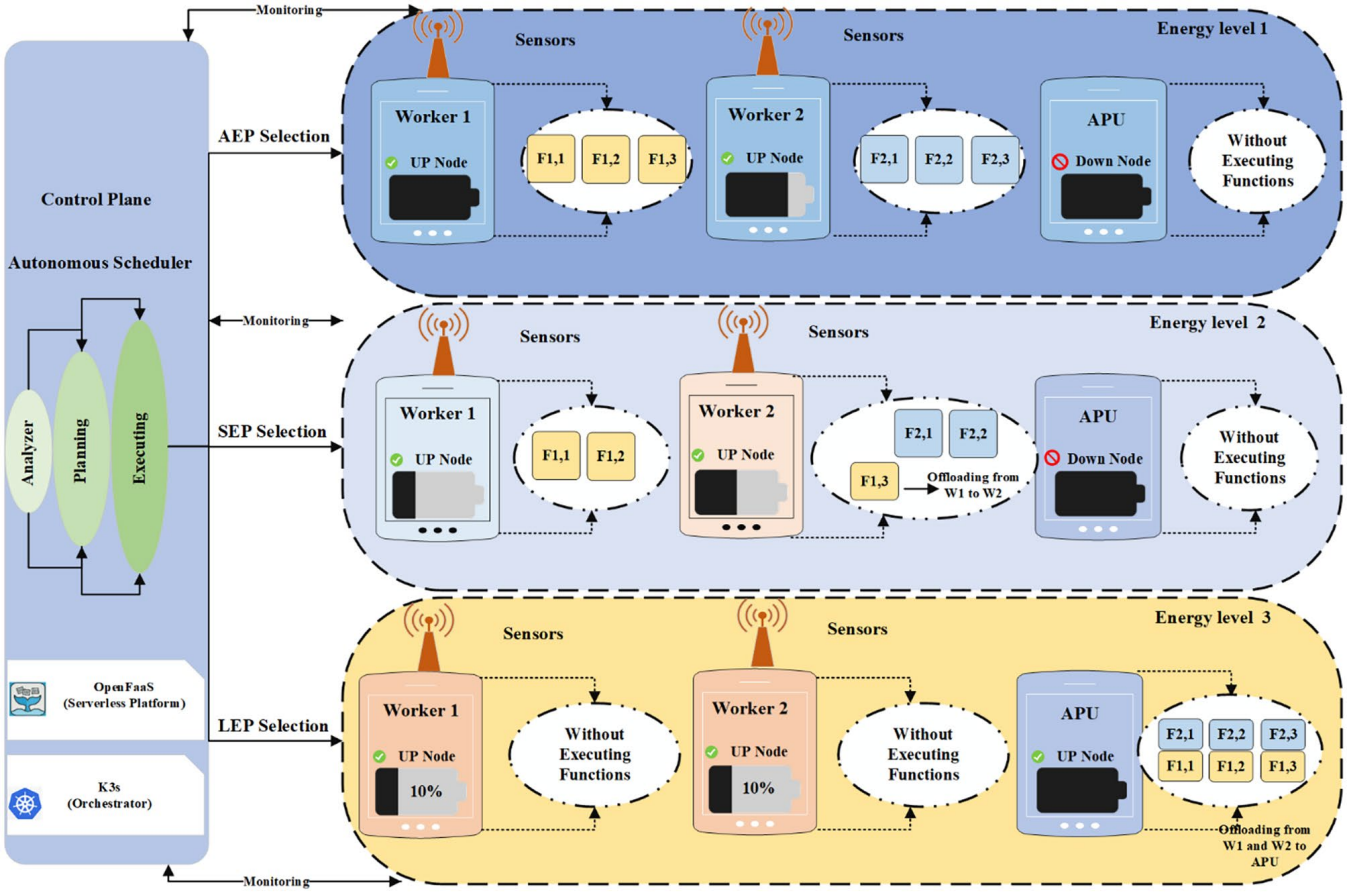
Overview of the proposed system architecture leveraging the MAPE‑based control loop.

Serverless computing has become a popular paradigm in the cloud, allowing developers to run code without the need to provision or maintain servers. In this model, cloud service providers handle all infrastructure concerns, allowing developers to focus solely on writing and configuring applications. Hence, this leads to increased productivity, accelerated development cycles, and reduced operational costs. At the same time, the proliferation of Internet of Things (IoT) devices has increased the need to process data closer to its source^1^. Edge computing solves this problem by performing computations at or near IoT endpoints, thereby reducing latency and improving performance over sending each data packet to central data centers. Together, these two approaches create serverless at the edge, where functions and services run alongside devices without the need to manage servers^2^. One of the main obstacles in edge-based serverless deployments is the limited resources of IoT hardware and their power budget constraints. Many endpoints have limited computing and storage capabilities and must optimize energy consumption to extend device lifetime and reduce costs. Energy-aware schedulers offer an effective solution: by continuously monitoring available and consumed power, they dynamically assign tasks to nodes in a way that balances the workload and conserves energy^3^. In addition to energy optimization, managing sudden incoming requests and ensuring failure resistance, especially when a scheduler algorithm fails, are critical challenges, as ignoring these issues can degrade overall system performance, increase costs, and reduce user satisfaction^4^. To address these issues, we propose a framework using the IBM Autonomic Computing model and an MAPE-based control loop to coordinate multiple energy-aware schedulers at the network edge. First, the framework monitors each node’s remaining energy and request load. Then, it estimates the power requirements for queued functions and analyzes the network state. Based on this analysis, an appropriate scheduler is selected and implemented. This loop is executed periodically and continuously adapts to changing conditions. We evaluated our AEAS by comparing its energy consumption and latency performance against traditional load-balancing and scheduling strategies. We have demonstrated significant improvements in efficiency and device lifetime. The framework is illustrated in Fig. 1.

With the increasing growth of serverless computing and widespread Internet of Things applications at the network’s edge, systems have faced significant challenges. These challenges include (1) limited energy resources at edge nodes, (2) severe workload fluctuations and instantaneous changes in energy levels, (3) the need to allocate resources optimally without increasing management overhead, and (4) maintaining availability and rapid response even under resource depletion conditions. In many sensitive edge network applications, such as smart cities and homes, remote healthcare, and environmental monitoring, these problems can lead to reduced system efficiency, increased response latency, and network instability. Therefore, it is important to provide solutions that can make optimal and automated decisions in resource management in the face of these dynamic conditions. In this regard, this study introduces an energy-aware pre-scheduling mechanism that allocates resources optimally for executing serverless functions by analyzing the energy levels of active nodes in real time. The scheduler selection problem based on the network energy state is formally defined, and a dynamic framework is designed to select the most appropriate scheduling strategy at any moment. Also, the performance of the proposed framework is evaluated under different workload distribution patterns, and experimental results have shown that this method significantly improves efficiency and energy effectiveness in edge environments. The remainder of this paper is structured as follows: Section “Background” provides the background necessary to understand the problem domain; Section “Related works” reviews related works in the field; Section “Framework for the proposed model” introduces the framework of the proposed model; Section “Formulation of the problem” formally defines the problem formulation; Section “Proposed algorithm” describes the proposed algorithm in detail; Section “Evaluation of performance” presents and discusses the performance evaluation results; and Section “Conclusion” concludes the paper and suggests directions for future research.

## Background

This section introduces the concept of serverless edge computing and provides a brief overview of the types of scheduling used in this context.

### Edge-based serverless architecture

**Fig. 2:**
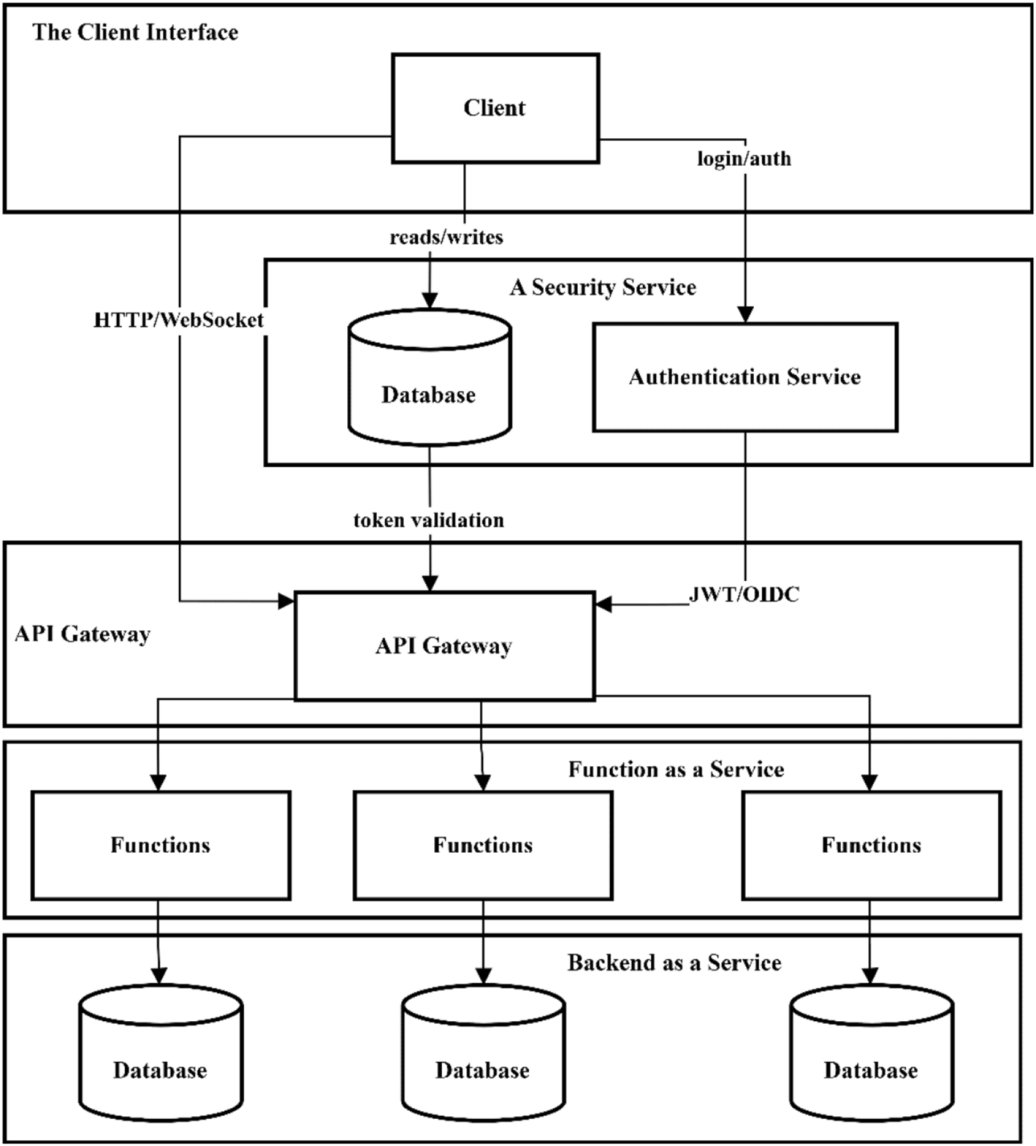
Serverless computing architecture overview.

Serverless computing is a model in which the cloud service provider manages the server infrastructure, and developers focus only on the application code without worrying about server configuration and maintenance. This approach, with automatic scalability and pay-as-you-go, increases developer productivity and reduces costs. Its applications include web and mobile app development, big data analysis, and data management in IoT. Serverless high compatibility with these areas is due to its good performance and high flexibility, and its fundamental concepts play a key role in this efficiency^5^. Figure 2 shows the architecture of the model.

- *Client Interface*: The front-end layer comprises single-page applications distributed with a CDN at the edge to minimize data transfer latency. These clients manage user state with BaaS SDKs and send HTTP/WebSocket requests to the API Gateway^6^.
- *Security Service*: Authentication/authorization services such as AWS Cognito, Auth0, and Okta implement OAuth2 and OpenID connect to handle registration, login, and JWT issuance/renewal; this layer ensures secure access with IAM rules and RBAC/ABAC policies, and supports event monitoring with MFA, security logging, and SIEM integration^7^.
- *API Gateway*: API Gateway is a unified entry point for REST and GraphQL that routes requests to FaaS functions or microservices with routing, throttling, CORS, and schema validation. It provides features such as internal caching, versioning, payload transformation, and distributed authentication and plays a key role in securing and optimizing the data path^8^.
- *Function as a Service (FaaS)*: AWS Lambda, Azure Functions, or Google Cloud Functions environments run event-driven code components in isolation and auto-scale; their cost is calculated based on the number of function executions and execution time. FaaS functions support a variety of HTTP, Queue, Timer, or EventBridge triggers and monitor the status of cold starts and performance metrics with monitoring tools^9^.
- *Backend as a Service (BaaS)*: Platforms like Firebase, AWS Amplify, or Parse offer features like managed NoSQL/SQL databases, storage, real-time sync, cloud functions, and push notifications without server management. These services include client SDKs, offline data persistence, field-level security rules, and integrated user analytics dashboards to accelerate app development^10^.

Each component of the serverless model allows developers and administrators to benefit from its benefits by increasing efficiency and productivity. The IoT network reduces latency and the need for high bandwidth, and improves response time by collecting and exchanging data and using edge computing near sensors and devices.

### Scheduling strategy in serverless computing

**Fig. 3:**
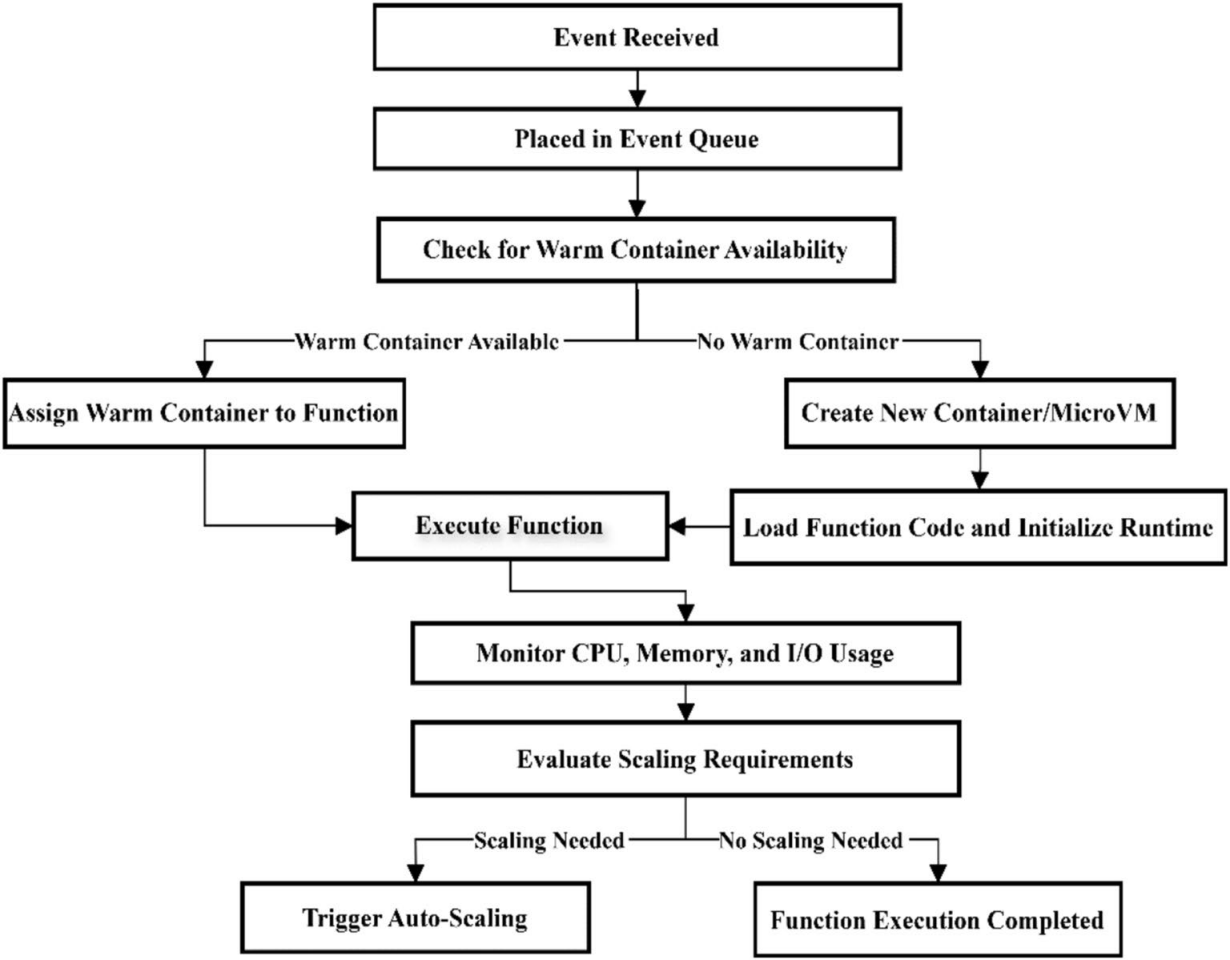
Operational flow of the scheduler in serverless computing environments.

In serverless computing architecture, scheduling is a multi-stage and optimized process that includes event queuing, decision-making about computing resource allocation, and function execution lifecycle management. The Scheduler allocates the most appropriate execution environment (Container or MicroVM) for each event by monitoring the system load, concurrency throttling policies, warm pool capacity, and vertical and horizontal scalability policies. This process is implemented by prioritizing requests (Priority Queuing), applying cost-time optimization (Cost-Time Optimization), and utilizing proactive provisioning strategies to reduce cold start time and optimize resource consumption, ensuring that the system’s responsiveness to dynamic changes in input rates is maintained with minimal latency and maximum efficiency^11^. Figure 3 illustrates the scheduler mechanism.

## Related works

In this section, we survey the state of the art in aware scheduling within cloud environments, spotlighting breakthroughs and persistent obstacles. By critically examining existing literature, we uncover gaps in current methodologies and suggest targeted research avenues to enhance the effectiveness and robustness of scheduling systems. Dayong and Dongzhi^12^ proposed a pod scheduling method based on serverless computing in the Kubernetes platform. They designed an algorithm for targeted resource allocation to schedule pods optimally. The implementation results show that this method significantly improves the efficiency of the scheduling process in Kubernetes and effectively manages resources. Li et al.^13^ proposed a method for optimal and sustainable resource allocation in fog computing environments, considering cost and QoS. This method uses the QoS status and the node reputation evaluation mechanism. The results showed that the proposed algorithm reduces cost by 20.9%, delay by 15.7%, and energy consumption by 12.6%, respectively, and increases system stability within QoS constraints. Zhang et al.^14^ proposed a VPaaS framework to process videos at the lowest possible cost. In this regard, cloud computing was used to provide the required resources. The results showed that prioritizing servers in the cloud layer during scheduling reduced response time and improved performance. Li et al.^15^ designed the MSTEC algorithm to reduce latency and increase reliability in task scheduling in fog environments, considering energy consumption constraints. This algorithm reduced the task execution time by an average of 16.5%. Also, to increase reliability in the presence of mobile nodes, the HREC algorithm was presented, which increased the system stability by 22% compared to MSTEC. Cheng and Zhou^16^ presented a framework called ARS for automatic resource scheduling in performance-as-a-service technologies. This framework is designed to manage unpredictable streaming data and automatically allocate the required resources by analyzing real-time data in streaming processes. Despite extensive research in energy-aware scheduling, resource optimization, and response time reduction, challenges such as variable energy resource management, limitations in increasing network availability, neglect of failure resistance components, and lack of proper QoS guarantees remain. These shortcomings prompted us to propose a new approach based on predictive models and the MAPE framework. This method aims to optimize energy consumption, increase the availability of active nodes, enhance failure resistance, and maintain an acceptable level of QoS in dynamic and distributed environments. Table 1 provides a comprehensive overview of previous studies on scheduling optimization and resource allocation in various computing environments. The table shows the type of approach, advantages, disadvantages, limitations, and year of publication of each study. This review aims to provide a clear picture of the progress of scheduling strategies, improving energy efficiency, and reducing latency in recent years.

**Table 1:** Comparison of approaches for optimizing resource allocation.

Refs.	Employment approach	Advantages	Limitations	Years
^12^	Energy	Increase operational capacity and the capacity to respond to requests in a reasonable time	The failure to use the proposed architecture when faced with various dynamically changing requests	2020
^13^	QoS	Adaptive placement cuts costs and latency Credibility‑based scheduling boosted node stability	Extra overhead from QoS‑state monitoring Performance hinges on accurate violation‑rate estimates	2023
^14^	Resource	Able to reduce the amount of bandwidth usage, along with reducing the round-trip time of requests	Failure to handle privacy concerns	2021
^15^	Energy	Achieves ~ 16.5% faster workflow completion Enhances system reliability by ~ 22%	Incurs overhead from energy-constrained monitoring Mobile node dynamics can still cause failures	2024
^16^	Resource	Able to best implement the real-time stream processing process by automatically allocating resources	Failure to address privacy issues Lack of assessment of the proposed mechanism for workloads created diversely and dynamically	2018
Proposed	Energy	Ensuring adequate resource provisioning for incoming tasks Extending the operational lifespan of active nodes in the network	Neglecting cybersecurity considerations Lack of integration of the proposed method with renewable energy strategies	-

## Framework for the proposed model

This section outlines a MAPE-based control loop framework underpinning an AEAS, followed by a formal statement of the problem.

### Self‑adaptive energy‑aware scheduling framework

**Fig. 4:**
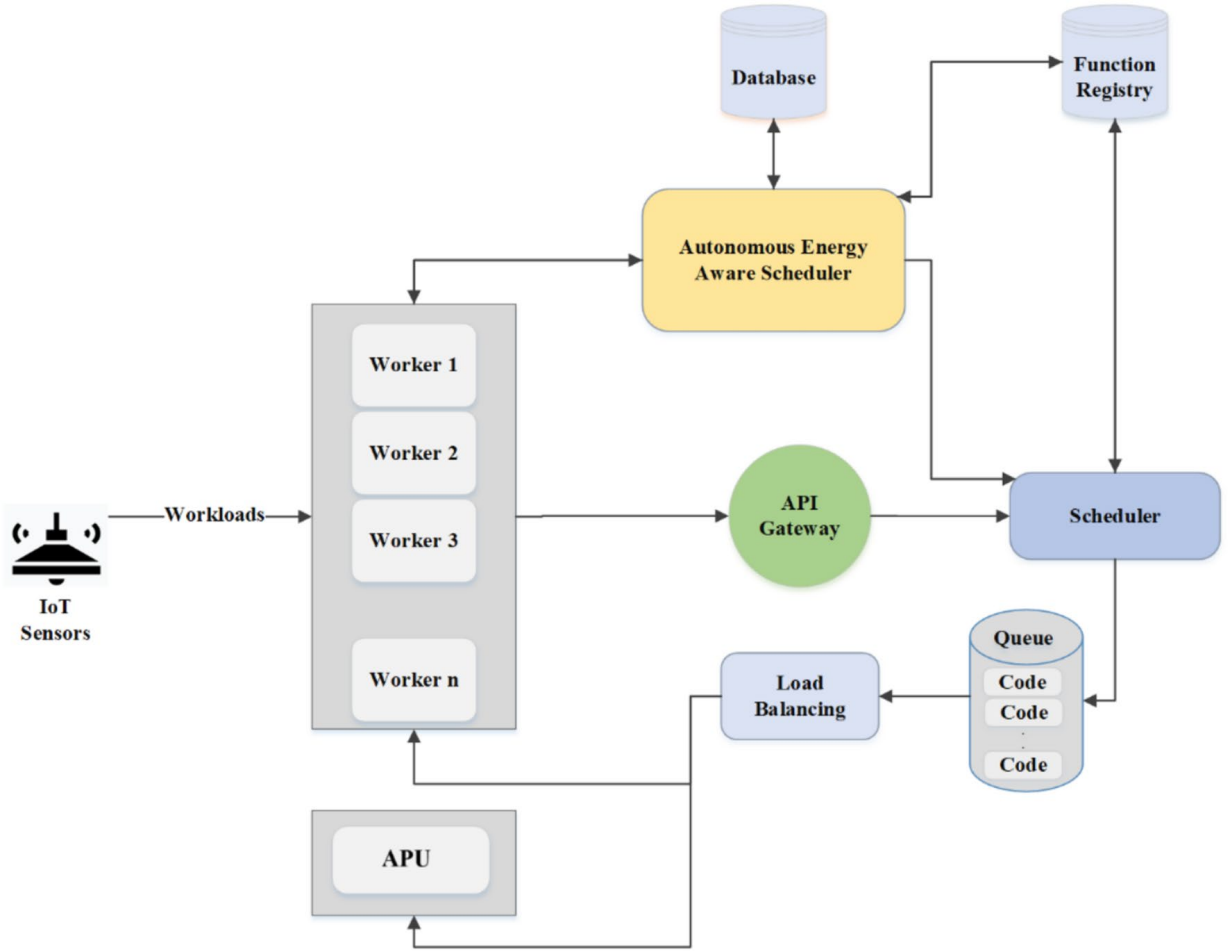
Energy-aware scheduling framework for complete program execution in serverless environments.

Figure 4 shows the workflow of the AEAS framework in the form of a four-stage MAPE cycle as follows: First, IoT sensors send their workloads to the API Gateway; at the same time, the monitoring module receives the battery energy level of the active nodes by querying and, by referring to the Function Registry, extracts the list of functions required to process those workloads. In the analysis phase, this data is converted into two separate lists (the remaining energy and energy required to execute the functions). Then, in the planning phase, the average energy available in the network, the average energy consumed by the calculation functions, and the health of the existing scheduling algorithms (which are different for different energy states) are checked and based on the ratio of available energy to required energy and the health status of each algorithm, the most appropriate Scheduler is selected and a certificate is issued including the Scheduler type and the network energy level. During the execution phase, Kubernetes activates the selected algorithm, and the selected Scheduler uses a FIFO queue and a Load Balancer to allocate functions to nodes that can execute them. In case of a power shortage on active nodes, the additional load is referred to the auxiliary processing unit (APU) node to keep the network available without interruption. This cycle is repeated at the end of each time interval so that the scheduling policy is always in sync with energy and workload changes and is optimal.

In this AEAS architecture, all components interact continuously in an MAPE loop to optimize the energy consumption and workload of the nodes. In the input layer, Workers execute computational tasks (Tasks) and at the same time, the Monitoring module, consisting of two subsystems Worker Monitoring and State of Battery Monitoring, collects real‑time information about the workload of each node and its battery charge over some time of $\Delta t$. This raw data is then sent to the Analyzer component called Workload & State of Battery Analyzer; here, while analyzing the required functions in detail (with the help of Function Registry), the actual amount of energy consumed (Estimated Energy Consumption) and the battery level of the active nodes (Active Node Energy Level) are estimated. The results are stored in the Database. After analysis, the Planning component calculates the average energy required by the functions (Average Required Energy) and the average usable energy in the nodes (Average Available Energy) by accessing the analyzed data and the information stored in the Database. Based on these calculations, the three scheduling strategies, AEP, SEP, and LEP, are evaluated in terms of efficiency and energy consumption. The most suitable Scheduler is selected, and a textual or structured Certificate is generated and recorded in the Database. This Certificate guarantees that the decision is based on the current network conditions and energy consumption goals. Finally, the Executor component (with the Apply Scheduler task) generates the necessary commands based on the issued Certificate and, if not available, based on the Default Scheduler and sends them to the execution queue so that the main system scheduler can apply the final commands to the nodes. This cycle is designed so that the output of each phase is the input of the next phase and is repeated continuously; in this way, AEAS can dynamically respond to instantaneous changes in workload and energy levels and ensure that the system’s energy consumption always remains optimal.

## Formulation of the problem

This section describes the markings and equations used to implement the proposed framework. Table 2 presents the essential parameters for solving the problem, as illustrated in Fig. 4. In this approach, time is segmented into uniform intervals, as defined in Eq. (1):

1 $${\mathcal{T}} = \left\{ {t_{k} \left| {t_{0} = 0,t_{k + 1} - t_{k} = \Delta t,t_{k} \in [0,T]} \right.} \right\}$$

**Table 2:** Notations and definitions.

Notation	Definition	Notation	Definition
$T$	Converts time into equal intervals	$\Xi$	Average energy of active nodes
$\mathcal{N}$	Active node count	$\beta$	Average processing energy required
$S$	The number of sensors per node	${\mathbb{C}}$	Net energy of internal active nodes
$\Lambda$	Sensor-generated workload rate on active nodes	$AEP$	Scheduler for high network energy vs. function demand
${\lambda }_{i,j}$	Workload rate $\Lambda$ from sensor $j$ in node $i$ at time $t$	$SEP$	Scheduler for an energy-deficient network scenario
$\Phi$	Number of functions needed per sensor load	$LEP$	Scheduler for a fully drained network scenario
${\phi }_{i,j}$	Function $\phi$ to process load from sensor $j$ in node $i$ at time $t$	${\pi }_{AEP}$	Check the $AEP$ scheduler’s health status or availability
$\varepsilon$	Energy level of active nodes’ batteries	${\pi }_{SEP}$	Check the $SEP$ scheduler’s health status or availability
${e}_{i}(t)$	The amount of energy $p$ in the battery of node $a$ in the time interval $t$	${\pi }_{LEP}$	Check the $LEP$ scheduler’s health status or availability
$\Psi$	Total energy in active nodes’ batteries	$C$	Certificate of selected scheduler
$\Omega$	Number of functions for sensor workloads	$CAEP$	The generated certificate is used to select the AEP scheduler
$\Pi$	Total energy required for functions	$CSEP$	Certificate for SEP scheduler selection
${\epsilon }_{i,j}^{a}$	Energy ε required to execute function $a$ by sensors $j$ in node $i$	$CLEP$	The generated certificate is used to select the LEP scheduler
${\epsilon }^{\Delta }$	Node with abundant resources	$CDS$	Certificate for DS scheduler selection
$minEnergy {\epsilon }^{\Delta }$	Minimum energy to sustain a redundant node	$DS$	Default Scheduler
$\mu$	Average minimum battery energy indicates active nodes	$\aleph$	Selected scheduler by AEAS
${\epsilon }^{a}$	Minimum battery power for an active node	${E}^{AEP}$	Energy consumption of all nodes in the $AEP$ scheduler
$\theta$	The maximum capacity that a resource can have	${E}^{SEP}$	Energy consumption of all nodes in the SEP scheduler
$\gamma$	Electricity consumption rate	${E}^{LEP}$	Energy consumption of all nodes in the LEP scheduler
${\vartheta }^{tx}$	Node $\vartheta$ energy consumption in $t$	${E}_{IAOA,HBI,BBO}$	Energy Loss Proportion by Strategy
${\vartheta }^{rx}$	Total energy consumed for overhead/functions	${R}_{IAOA,HBI,BBO}$	Rounds per strategy
$max\aleph_{{\tau \in \left\{ {AEP,SEP,LESP} \right\}}}$	Maximum scheduler availability across time frames	${Up}_{IAOA,HBI,BBO}$	Uptime of strategies

In this equation, time is divided into equal intervals where each interval has a duration of $\Delta t$. The scheduler updates active nodes each interval, defining the cluster as ${\mathcal{N}}$. This is shown in Eq. (2).

2 $${\mathcal{N}} = \{ n_{i} \left| {i = 1, \ldots ,k} \right.\}$$

In this equation, ${\mathcal{N}}$ is the set of nodes in the cluster, ${n}_{i}$ represents each node, and $i$ ranges from 1 to $k$.Edge nodes connect to surrounding IoT sensors, forming the set ${\mathcal{S}}$.This is shown in Eq. (3).

3 $${\mathcal{S}} = \{ s_{j} \left| {j = 1, \ldots ,d} \right.\}$$

In this equation, ${\mathcal{S}}$ represents the full sensor collection, ${s}_{j}$ each individual sensor, and $j$ ranges from 1 to $d$. Each sensor $j$ on node $i$ produces a time-dependent workload; collecting these nonnegative rates across all nodes, sensors, and time intervals gives the complete workload set.

This is shown in Eq. (4).

4 $$\Lambda = \left\{ {\lambda_{i,j} (t)\left| {i = 1, \ldots ,k,j = 1, \ldots ,d,t \in T,\lambda_{i,j} (t) \ge 0} \right.} \right\}$$

In this equation, $\Lambda$ is the full workload set, $\lambda_{i,j} (t)$ denotes the rate from the sensor $j$ on node $i$ at time $t$, and the constraint $\lambda_{i,j} (t) \ge 0$ ensures all workloads are nonnegative. Therefore, each sensor may generate zero or positive workload, which is processed by a specific function assigned to that sensor-node pair over time, as shown in Eq. (5).

5 $$\Phi = \left\{ {\phi_{i,j} (t)\left| {i = 1, \ldots ,k,\;j = 1, \ldots ,d,\;t \in T} \right.} \right\}$$

In this equation, $\Phi$ represents the set of functions, where $\phi_{i,j} (t)$ is the function linked to the sensor $j$ on node $i$ at time $t$. Hence, to manage the system efficiently, it is first essential to calculate the total number of nodes operating within the network, as shown in Eq. (6).

6 $$\Gamma = \sum\limits_{i = 1}^{k} {x_{i} }$$

In this equation, $\Gamma$ is the total count of active nodes, where ${x}_{i}$ indicates whether the node $i$ is active (1) or inactive (0). The framework also evaluates the available energy at each node over time, ensuring it remains within the battery’s capacity limits. This is shown in Eq. (7).

7 $${\rm E} = \left\{ {e_{i} (t)\left| {i = 1, \ldots ,k,\;t \in T,\;0 \le e_{i} (t) \le \mu } \right.} \right\}$$

In this equation, ${\mathcal{E}}$ is the set of available energies, where $e_{i} (t)$ is the energy at the node $i$ during time $i$, bounded between 0 and the maximum capacity $\mu$ Hence, the system sums the available power across all active nodes during all time intervals to monitor overall energy usage, as shown in Eq. 8.

8 $$\Psi = \sum\limits_{t = 0}^{T} {\sum\limits_{i = 1}^{k} {q_{i} } } (t)$$

In this equation, $\Psi$ represents the total energy, where ${q}_{i}(t)$ is the available energy at the node $i$ during time $t$. The AEAS also calculates the number of functions needed to handle all sensor workloads across the network over time, as shown in Eq. (9).

9 $$\Omega = \sum\limits_{t = 0}^{T} {\sum\limits_{i = 1}^{k} {\sum\limits_{j = 1}^{d} {\phi_{i,j} } } } (t)$$

In this equation, $\Omega$ is the total number of processing functions, where ${\phi }_{i,j}(t)$ represents the function assigned to the sensor $j$ on node $i$ at time $t$. Hence, to organize the required processing functions, they are grouped into a set representing all needed functions throughout the system, as shown in Eq. (10).

10 $$\Theta = \left\{ {\theta^{i} \left| {i = 1, \ldots ,u} \right.} \right\}$$

In this equation, $\Theta$ is the set of all required functions, where $\theta^{i}$ denotes the $i$ the function, and $u$ is the total number of functions needed. Next, the system calculates the sum of processing energies to estimate the total energy needed for executing all requested functions, as shown in Eq. (11).

11 $$\Pi = \sum\limits_{a = 1}^{u} {\sum\limits_{i = 1}^{k} {\sum\limits_{j = 1}^{d} { \in_{i,j}^{a} } } }$$

In this equation, $\Pi$ represents the total processing energy, where $\in_{i,j}^{a}$ is the energy required to execute the $a$-th function for the sensor $j$ on node $i$. On the other hand, to assess the network’s energy health, the system computes the average of the minimum available energy across all nodes, as shown in Eq. (12).

12 $$\Upsilon = {\text{avg}}\left( {\frac{{\sum\nolimits_{i = 1}^{k} {{\text{minEnergy}}_{i} } }}{\Gamma }} \right)$$

In this equation, $\Upsilon$ represents the average minimum energy, where ${\text{minEnergy}}_{i}$ is the minimum energy of the node $i$ and $\Gamma$ is the total number of active nodes. Since maintaining node activity demands at least 10% of the battery, the network’s average usable energy is determined by subtracting this threshold, as shown in Eq. (13).

13 $$\Xi = \left( {\frac{{{\text{avg}}(\Psi )}}{\Gamma }} \right) - \Upsilon$$

In this equation, $\Xi$ is the network’s average net energy, where Ψ is the total energy across nodes and Υ is the average minimum required energy. The system calculates the average energy consumption across all requested functions to estimate the energy needed per function, as shown in Eq. (14).

14 $$\beta = {\text{avg}}\left( {\frac{\Pi }{\Omega }} \right)$$

In this equation, $\beta$ denotes the average energy needed to process one requested function, where $\Pi$ is the total processing energy and Ω is the total number of functions. The AEAS incorporates four scheduling modes based on available energy: AEP (enough energy for all tasks), SEP (partial energy sufficiency), LEP (insufficient energy), and DS (default scheduler if others fail). Before applying any schedule, its operational status must be validated using the following health checks, shown in Eqs. (15–17).

15 $$\pi_{{{\text{AEP}}}} = \left\{ {\begin{array}{*{20}l} 1 \hfill & {\quad {\text{if}}\, \, \eta \, \in \,{\text{AEP }}\,{\text{response}}} \hfill \\ 0 \hfill & {\quad {\text{otherwise}}} \hfill \\ \end{array} } \right.$$

In this equation, $\pi_{{{\text{AEP}}}}$ indicates whether the AEP scheduler is active (1) or inactive (0).

16 $$\pi_{{{\text{SEP}}}} = \left\{ {\begin{array}{*{20}l} 1 \hfill & {\quad {\text{if }}\,\eta \, \in \,{\text{SEP }}\,{\text{response}}} \hfill \\ 0 \hfill & {\quad {\text{otherwise}}} \hfill \\ \end{array} } \right.$$

In this equation, $\pi_{{{\text{SEP}}}}$ shows if the SEP scheduler successfully responds (1) or fails (0).

17 $$\pi_{{{\text{LEP}}}} = \left\{ {\begin{array}{*{20}l} 1 \hfill & {{\text{if}}\, \, \eta \, \in \,{\text{LEP}}\,{\text{ response}}} \hfill \\ 0 \hfill & {{\text{otherwise}}} \hfill \\ \end{array} } \right.$$

In this equation, $\pi_{{{\text{LEP}}}}$ reflects the responsiveness of the LEP scheduler (1 for active, 0 for inactive). The AEAS generates a certificate that defines the current network energy status and selects the appropriate scheduling strategy, as detailed in Eq. 18.

18 $$\aleph = \left\{ {\begin{array}{*{20}l} {{\text{CAEP}}} \hfill & {\quad {\text{if }}\Sigma < \Xi {\text{ and }}\pi_{{{\text{AEP}}}} = 1} \hfill \\ {{\text{CSEP}}} \hfill & {\quad {\text{else if }}\Upsilon < \Xi < \Sigma {\text{ and }}\pi_{{{\text{SEP}}}} = 1} \hfill \\ {{\text{CLEP}}} \hfill & {\quad {\text{else if }}\Xi \le \Upsilon {\text{ and }}\pi_{{{\text{LEP}}}} = 1} \hfill \\ {{\text{CDS}}} \hfill & {\quad {\text{otherwise if all }}\pi_{{{\text{AEP}}}} ,\pi_{{{\text{SEP}}}} ,\pi_{{{\text{LEP}}}} = 0} \hfill \\ \end{array} } \right.$$

In this equation, $\aleph$ Eq. (19) represents the scheduler certificate, which is selected based on energy conditions and the availability of AEP, SEP, or LEP schedulers. The final scheduler is chosen according to the generated certificate.

19 $${\mathbb{Q}} = \left\{ {\begin{array}{*{20}l} {{\text{AEP}}} \hfill & {\quad {\text{if }}\Phi = {\text{CAEP}}} \hfill \\ {{\text{SEP}}} \hfill & {\quad {\text{else if }}\Phi = {\text{CSEP}}} \hfill \\ {{\text{LEP}}} \hfill & {\quad {\text{else if }}\Phi = {\text{CLEP}}} \hfill \\ {{\text{DS}}} \hfill & {\quad {\text{otherwise if }}\Phi = {\text{CDS}}} \hfill \\ \end{array} } \right.$$

In this equation, ${\mathbb{Q}}$ identifies the chosen scheduler based on the corresponding certificate Φ. The Scheduler Selector (SS) ensures network operation even under critical low energy by picking the best scheduling strategy. Equation (20) computes the net available energy for optimal selection.

20 $$\Delta = \in^{\Delta } - {\text{minEnergy}}^{\Delta }$$

In this equation, Δ represents the energy surplus for active nodes compared to their minimum required energy.

### Operational constraints

#### Constraints 1

Only active nodes are considered for assessing network energy and selecting a scheduler, while APU resources are excluded from these calculations, as expressed in Eq. 21.

21 $${\rm P} = \left( {\sum\limits_{t = 1}^{T} {\sum\limits_{a = 1}^{k} {e_{t}^{a} } } - e^{\delta } } \right) - \left( {\sum\limits_{a = 1}^{k} { \in_{a} } - \in^{\delta } } \right)$$

In this equation, P represents the adjusted network energy, where the APU resource nodes’ energy $e^{\delta }$ and minimum charge $\in^{\delta }$ are subtracted from the total values.

#### Constraints 2

The default scheduler (DS) is excluded from the energy-based selection since it operates independently of the network’s energy. It is only activated if other schedulers are unavailable, as shown in Eq. (22).

22 $${\mathbb{C}} = \left\{ {\begin{array}{*{20}l} {\{ {\text{AEP}},{\text{SEP}},{\text{LEP}}\} } \hfill & {\quad {\text{if }}\,{\text{CAEP}},\,{\text{CSEP}},\,{\text{CLEP}}\,{\text{ are}}\,{\text{ valid}}} \hfill \\ {{\text{DS}}} \hfill & {\quad {\text{otherwise}}} \hfill \\ \end{array} } \right.$$

In this equation, ${\mathbb{C}}$ defines the chosen scheduling set based on the availability of energy-aware schedulers; otherwise, it defaults to DS.

#### Constraints 3

The energy available in an APU resource node can be equivalent to the total energy of all active nodes. If the active nodes cannot process the requested functions, the APU node must have sufficient resources to handle the requests, as illustrated in Eq. (23).

23 $$\left( {\frac{{\sum\nolimits_{t = 1}^{T} {\sum\nolimits_{a = 1}^{k} {e_{t}^{a} } } }}{N} - \mu } \right) \le \delta$$

In this equation, the left-hand side represents the difference between the average energy per node and the minimum required energy, which must not exceed the threshold $\delta$ for the APU node to provide adequate support.

## Proposed algorithm

**Fig. 5:**
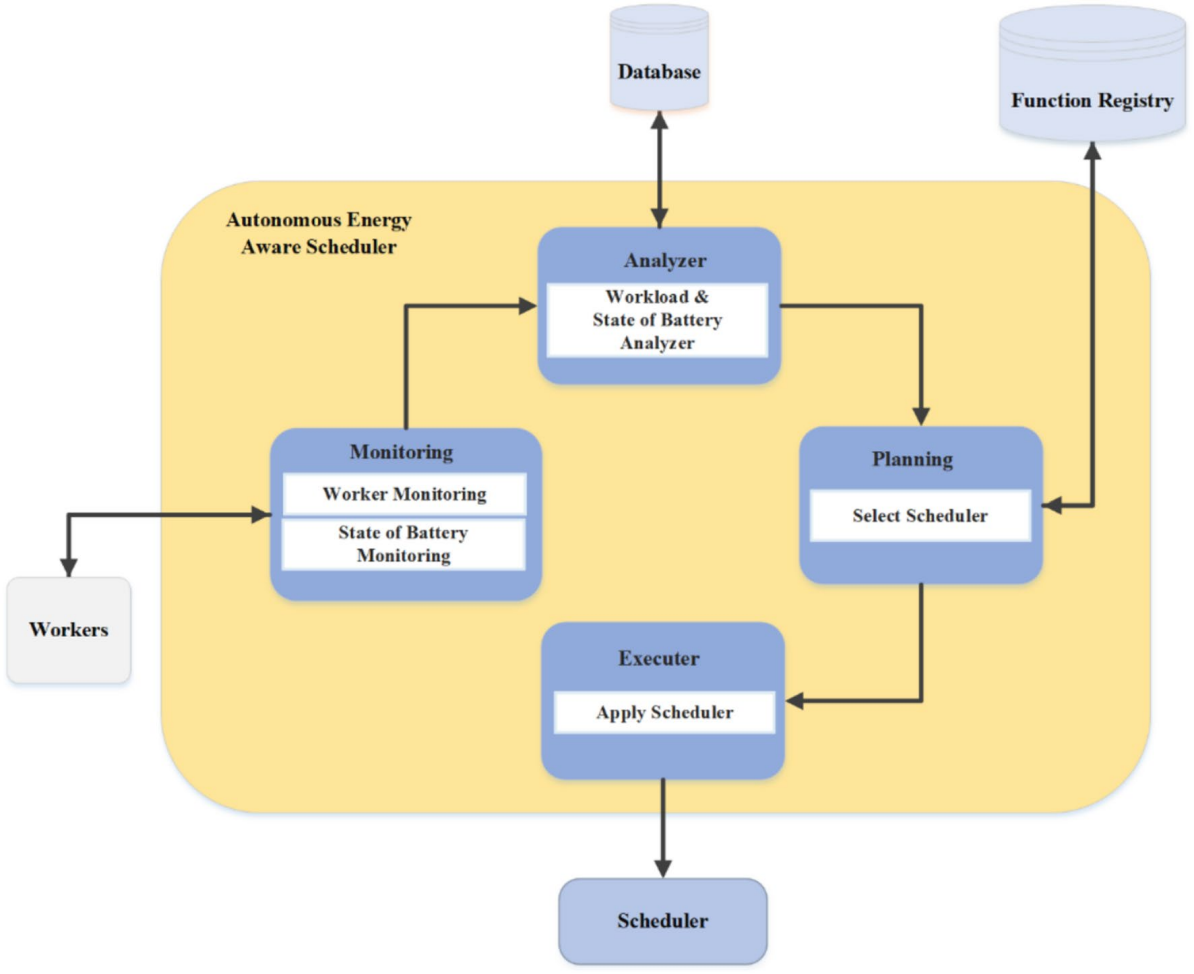
Structure of AEAS.

For self-adaptation of the AEAS framework based on the IBM Autonomic Computing model and optimal control of energy consumption, the MAPE loop has been used, which includes four phases: Monitor, Analyze, Plan, and Execute; In the Monitor phase (Fig. 5 and Algorithm 2), the workload status of each node and their battery energy level are monitored with a time interval of $\Delta t$. Then, in the Analyze phase (Fig. 5 and Algorithm 3), the collected data is processed, and the functions required for processing the load are identified. The containers are warm-started, and the exact energy level of the active nodes is estimated. In the Plan phase (Fig. 5 and Algorithm 4), by calculating the average energy required by the functions and the average energy available in the nodes and checking the correct operation of the AEP, SEP, and LEP Schedulers, the most appropriate strategy is selected and the corresponding “certificate” is issued and stored in the database. Finally, in the Execute phase (Fig. 5 and Algorithm 5), based on the certificate issued by one of the AEP, SEP, and LEP Schedulers or, if they do not exist, the Default Scheduler, tasks are activated and placed in the execution queue. This cycle is designed so that the output of each phase becomes the input of the next phase and is repeated continuously so that the framework can dynamically respond to changes in workload and energy.

**Algorithm 1:**
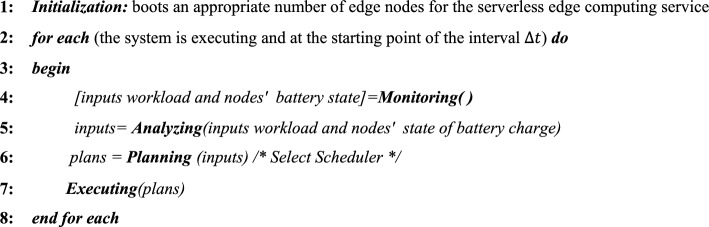
**Pseudo-code for AEAS**

### Monitoring Phase

In the Monitoring phase, in each interval $\Delta t$, first the amount and type of workloads ($\lambda$) generated by sensors $(s)$ in each node $(n)$ (Lines 3–5) are determined and processed, and then the energy level of the batteries of active nodes (except APU nodes) is measured (Lines 6–8). The output of this phase is complete knowledge of the type of workloads and the amount of energy of each node, which is used in the next phase (Line 10) to select the Scheduler.

**Algorithm 2:**
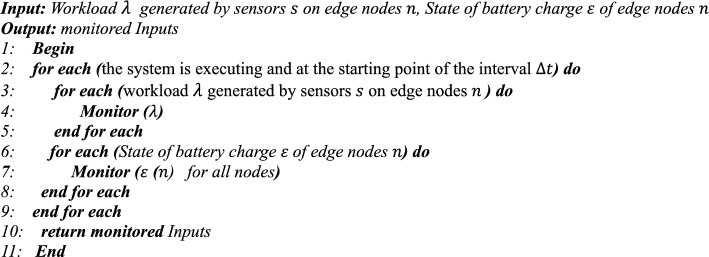
**Pseudo code for Monitoring Phase()**

### Analyzing phase

This step analyzes and processes the information collected in the Monitoring phase. First, Algorithm 3 examines the workload $\lambda$ generated by the sensors $s$ on the nodes $n$ and uses the information about them from the previous step (lines 4 to 6). Then, the type of functions required to process the workloads is identified by the function_identify function. Hence, to reduce the latency in calling the functions, the warm_start () function is executed before the scheduling process to send the necessary requests (line 7). In the next step, the energy available in the node’s batteries is calculated using the state_of_charge () function and stored in a list called state_of_charge () (lines 9 to 14). Also, the energy required for the nodes to be active must be greater than the specified minimum energy (lines 10 to 13). The output of this step is two lists: one containing the functions required to process the workloads and the other containing the amount of energy available in the network’s active nodes (line 16).

**Algorithm 3:**
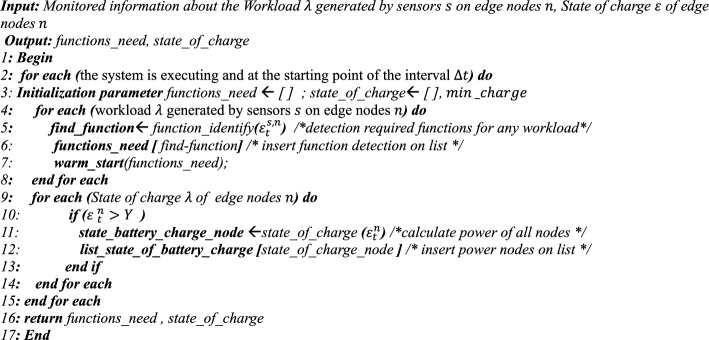
**Pseudo code for Analysis Phase()**

### Planning phase

Based on the information from the previous step, an attempt is made to select the appropriate Scheduler based on the network energy level. Here, a Certificate is generated that specifies the type of Scheduler and its health status. Algorithm 4 has two inputs, the same as the outputs of the Analyzing step, and are entered as a List. Hence, to process the List, the energy required to execute each function is first calculated, and then these values are summed (lines 3–7) and averaged (line 13). The same process is performed for the nodes, and the energy available in each node is calculated (lines 8–12), and then the average energy of the entire network is obtained (line 14). Hence, using the check_health_scheduler function, the health of the Schedulers is checked (lines 15–16). Considering criteria such as availability and compliance with the conditions, a Scheduler is selected, and a Certificate is issued for it (lines 17–25). The selected state is stored in the database via the sort_state_on_database function (line 27). If no other Schedulers are available, the Default Scheduler (DS), which is disabled, is activated.

**Algorithm 4:**
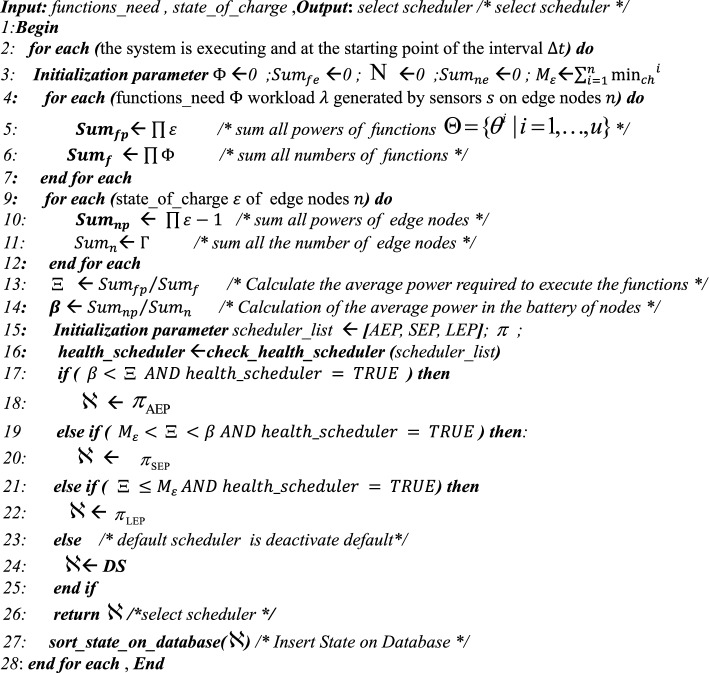
**Pseudo code for Planner Phase()**

### Executing phase

In the Execution phase, the decisions of the Planning phase are acted upon. The input to Algorithm 5 is the certificate generated in the Planning phase, which determines which Scheduler to run. If the certificate sent does not point to any Scheduler, it is assumed that all Schedulers are unavailable for some reason. In this case, the Default Scheduler, which was disabled, is enabled, and the system continues to work.

**Algorithm 5:**
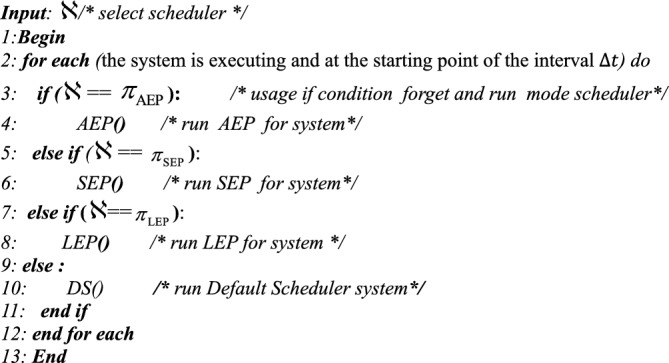
**Pseudo code for Execution Phase ( )**

## Evaluation of performance

In this section, the proposed mechanism is tested by being implemented under different workload generation patterns and scheduling algorithms. The following sections describe the implementation settings and the metrics evaluated, and discuss the obtained results.

### Experimental configuration

**Fig. 6:**
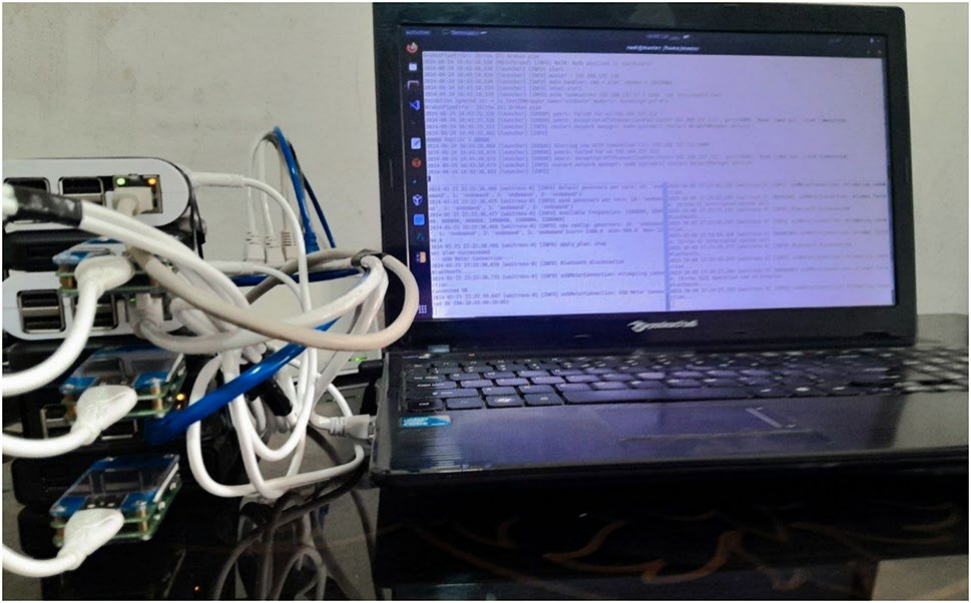
Design and implementation of a serverless edge computing cluster based on raspberry pi and LAptops.

The implemented framework consists of a star topology with four nodes connected via a switch and without an internet connection: a Packard Bell Easy Note TK85 laptop with a Core i5 processor, 4 GB RAM and 512 GB SSD as the control panel (master), two Raspberry Pi 3 B with a Quad-core ARM Cortex-A53 64-bit processor, 1 GB RAM and a 32 GB MicroSD card as worker1 and worker2, and a Raspberry Pi 4 B with a Quad-core ARM Cortex-A72 64-bit processor, 4 GB RAM and a 32 GB MicroSD card as the APU node (default disabled). The boards are powered by two Remax Jane Series power banks (model RPP‑119 with a capacity of 10,000 mAh and model RPP‑142 with a capacity of 20,000 mAh; both with DC 5V–2.1A input/output), and their energy consumption is measured using a UM25C power meter with a 1.44‑inch color LCD via Bluetooth. Network communication is established with a Tesco TNC510 CAT5 cable and a TP‑Link TL‑SF1005D V15 switch (5‑Port 10/100 Mbps). For development and implementation, Python 3.8 is used as the programming language, Docker version 20.10 as the CRI, and OpenFaaS as the serverless platform in a container orchestrator, and container management is performed with K3s v1.23 (lightweight Kubernetes) for IoT devices at the edge of the network. The AEAS has also been developed with Python; the implementation diagram is shown in Fig. 6.

**Fig. 7:**
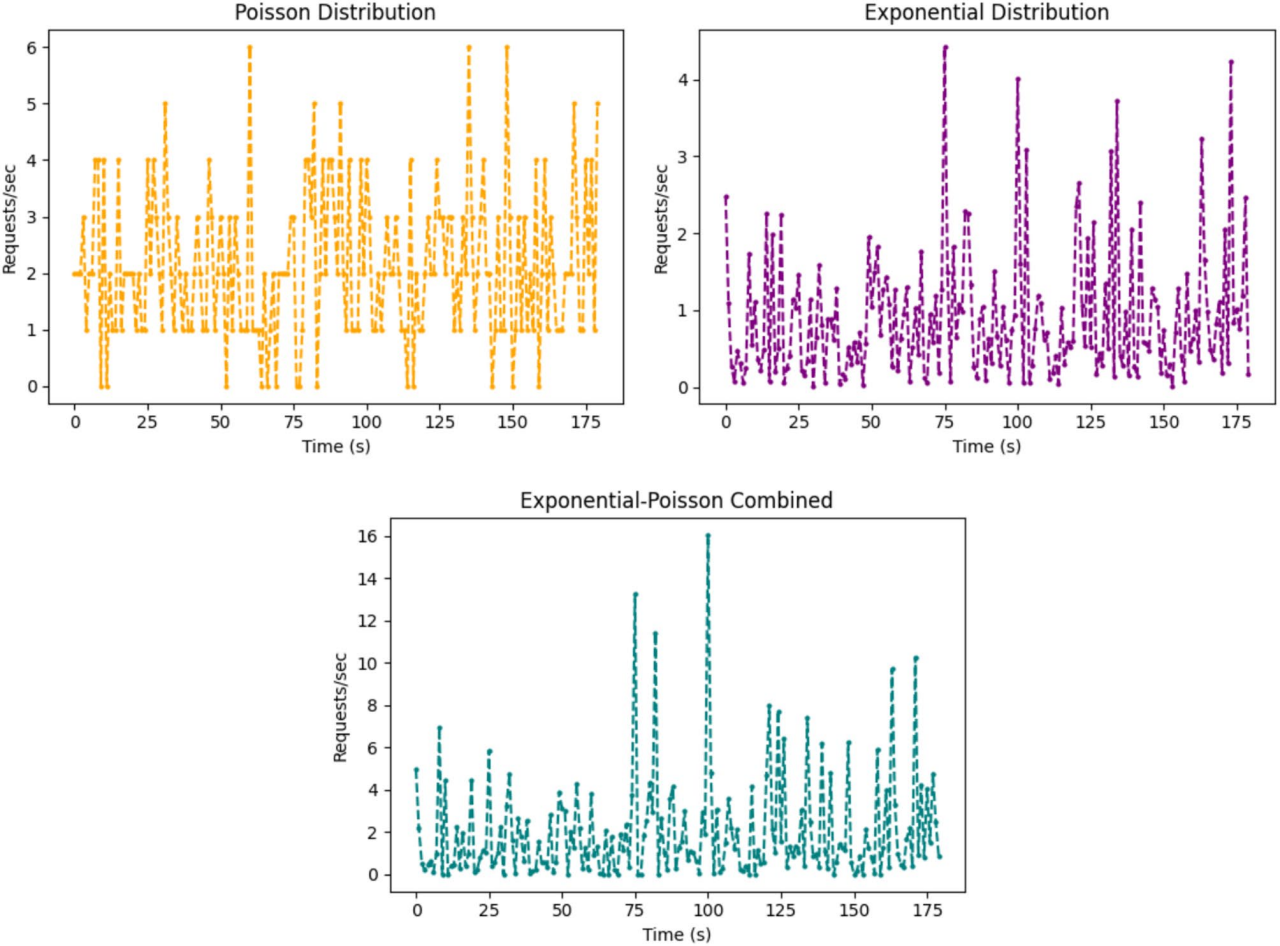
Workload distributed patterns: poisson, exponential, and exponential-poisson.

Figure 7 illustrates three load generation patterns for implementing and evaluating the model. For the evaluation, HTTP requests are transmitted to both active nodes (workers 1 and 2) for 120 s; each request contains one photo. Each round lasts 20 min, and five complete rounds are executed every two hours (without an APU). The number of rounds is 35 for both the reference and proposed methods, resulting in a total evaluation period of 14 h. The implementation reflects real-world conditions by simultaneously simulating two CCTVs in a home and sending 160 photos (if motion is detected). Finally, the suggested mechanism is compared with other scheduling methods under three workload distribution patterns. The poison distribution models the probability of a given number of events occurring in a given time or space interval based on the average rate of occurrence. This distribution is represented in Eq. (24)^17^.

24 $$P(N = n) = \frac{{\mu^{n} e^{ - \mu } }}{n!}$$

This equation gives the probability of exactly $n$ events occurring in an interval, where $N$ is a discrete random variable, $\mu^{{\text{n}}}$ is the expected number of events, $n!$ is the factorial of $n$, and e is Euler’s number (~ 2.71828). The Exponential-Poisson distribution is a hybrid load distribution model where the randomness of task arrival (modeled by a Poisson process) is combined with the variable processing time (modeled by an Exponential distribution). The combined load behavior is formulated in Eq. (25)^18^.

25 $$EP_{i} = \lambda_{i} \cdot \beta_{i} \cdot R_{i}$$

In this equation, ${EP}_{i}$ represents the expected load on the resource $i$, where ${\lambda }_{i}$ is the Poisson arrival rate, ${\beta }_{i}$ is the average processing time, and ${R}_{i}$ indicates resource availability. The exponential distribution is one of the most important distributions in probability theory and statistics. It is used to estimate the time interval between successive events in a process. This distribution is represented in Eq. (26)^19^.

26 $$f(\rho ) = \lambda {\mkern 1mu} e^{ - \lambda \rho }$$

This equation represents the probability density of observing an event at time $\rho$, where $\lambda$ is the rate parameter (average occurrences per unit time), and e is the natural logarithm’s base, controlling the density curve’s exponential decay.

### Evolution metrics

In this section, the AEAS’s performance is examined based on evaluation criteria, including failure resistance, uptime, and energy expenditure, to confirm the achievement of the research objectives.

- *Failure Resistance (RF)*: This evaluation parameter is implemented by introducing an independent scheduling structure. First, each scheduler’s availability is checked, and network energy level and health status are confirmed by issuing a certificate. If any scheduler is disabled, the structure automatically selects a suitable replacement through a self-healing process and recovers the system according to Eq. (27).
  27 $$\aleph_{RF} = \left\{ {\begin{array}{*{20}l} {SEP,RecAEP\quad if\quad CAEP\left( { HAEP = 0} \right)} \hfill & {} \hfill \\ {AEP,RecSEP\quad if\quad CSEP\left( { HSEP = 0} \right)} \hfill & {} \hfill \\ {LEP,RecAEP,RecSEP\quad if \quad CAEP\left( { HAEP = 0} \right)} \hfill & {\quad AND CSEP\left( { HSEP = 0} \right)} \hfill \\ {DS ,RecAEP,RecSEP,RecLEP\quad else} \hfill & {} \hfill \\ \end{array} } \right.$$
- *Uptime*: In the AEAS framework, considering the workload size and network energy level, the most optimal scheduler is selected to reduce system access time and enable the processing of various workloads even under energy shortage conditions. As a result, the operational availability of the scheduler and active nodes is maximized in Eq. (28).
  28 $$\max \aleph_{{\tau \in \{ AEP,{\kern 1pt} SEP,{\kern 1pt} LEP,{\kern 1pt} DS\} }} \sum\limits_{t = 0}^{T} [ (\min (\tfrac{{\overline{\psi } }}{\Gamma }{ - }\overline{S} ) + \delta ){ - }\max (\tfrac{{\overline{\Pi } }}{\Omega })]^{\tau }$$
- *Energy Consumption*: The AEAS is structured to choose and run appropriate schedulers according to the network’s energy conditions. Its goal is to optimize resource usage and energy efficiency. It incorporates three distinct schedulers, each utilizing a unique strategy. AEP: The network is assumed to have enough energy to handle all incoming requests, so the processing occurs on active nodes. SEP: When the network’s energy is insufficient to process all requests, the remaining unprocessed requests are offloaded to a node equipped with APU resources to preserve system responsiveness. LEP: If the network’s energy level drops to a minimum, all requests are redirected to a node with adequate resources to ensure the system remains responsive. The corresponding calculation is given in Eq. (29).
  29 $$E^{AEP,SEP,LEP} = \sum\limits_{i = 1}^{n} {\left( {\frac{{d_{i}^{t} }}{\theta } \times \gamma \times (\vartheta^{tx} + \vartheta^{rx} ) \times \delta t} \right)}$$

In this equation, $\frac{{d}_{i}^{t}}{\theta }$ represents the energy directly consumed based on the measured usage or occupation of resources. This value is then multiplied by the power consumption rate $\gamma$ in the following step.

### Analysis of results

We design a set of experiments under different scenarios to assess the efficiency of the proposed mechanism. We compare the proposed scheduling algorithm with three main scheduling approaches across three different workload distribution patterns in these experiments. The descriptions of these three scheduling approaches are provided below.

- *Arithmetic optimization algorithm (IAOA):* This approach uses an improved version of the IAOA that provides faster and more accurate convergence by strengthening the search operators. Hence, the algorithm optimizes user equipment (UE) task offloading decisions and MEC server resource allocation by modeling energy consumption under time and constraints^20^.
- *Hybrid Bio-Inspired (HBI)*: A hybrid Bio-Inspired algorithm combining Modified PSO and CSO is proposed for task scheduling and resource management in a cloud environment. First, task assignment to virtual machines is optimized using the Modified PSO algorithm, and then resource management is performed according to each task’s requirements through a hybrid algorithm^21^.
- *Biogeography-based optimization (BBO):* This approach, based on the BBO algorithm, is presented as a multi-objective strategy for task offloading in MEC. The BBO algorithm aims to solve the multi-criteria optimization problem, replacing traditional methods and optimizing the decision-making process in complex and multi-objective environments^22^.

In this study, three situations are designed: in the Situation 1 using Workloads, Poisson, Exponential and Exponontial‑poisson distributions, IAOA, HBI, BBO, and the proposed method in Energy CONSUMPTION and Energy Waste Reduction; In Situation 2 without determining the type of workloads, the system Failure Resistance is evaluated when the schedulers are unavailable; And in the Situation 3, the same Workloads is the Network Uptime duration of the algorithms.

#### Situation 1: Enhanced energy efficiency (Decreasing energy wastage)

**Fig. 8:**
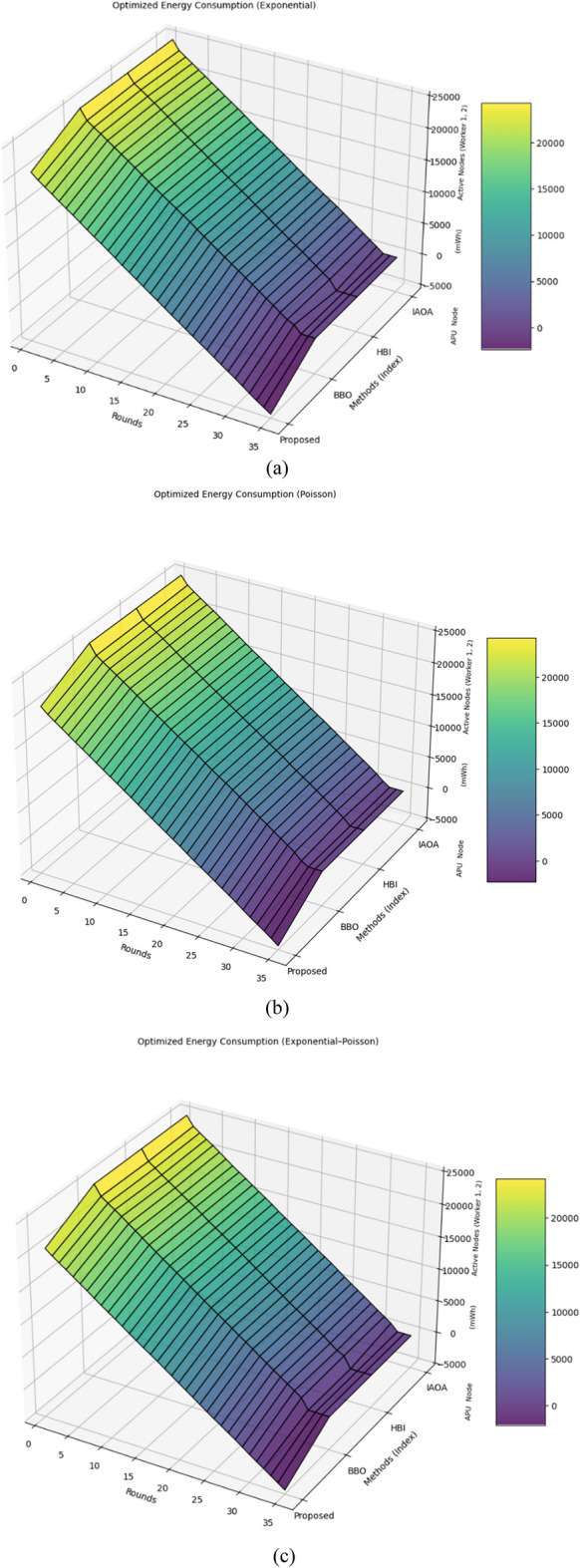
Assessment of consumed energy across workload types: Exponential (**a**), Poisson (**b**), and Exponential-poisson (**c**).

The data in Table 3 demonstrate that our proposed mechanism consistently achieves the highest enhanced energy efficiency (decreasing energy wastage) across all load‑distribution models: exponential, Poisson, and Exponential–Poisson outperforming IAOA, HBI, and BBO. For the exponential model, IAOA (32 rounds) wasted 1.67% (486 mWh), HBI (31 rounds) wasted 1.99% (530 mWh), and BBO (32 rounds) wasted 1.33% (430 mWh). In contrast, our method incurred zero wastage over 35 rounds by guaranteeing full execution of every task (see Fig. 8a). Likewise, in the Poisson scenario (32 rounds), the wastage for IAOA, HBI, and BBO was 1.71% (490 mWh), 1.81% (508 mWh), and 1.63% (481 mWh), respectively (Fig. 8b), and in the Exponential–Poisson case (32–31 rounds) those figures were 1.40% (440 mWh), 1.59% (460 mWh), and 1.79% (500 mWh) (Fig. 8c). By continuously monitoring IoT devices’ energy reserves and treating any partial-function failure as a complete process failure, our approach eliminates incomplete executions, boosting overall enhanced energy efficiency (decreasing energy wastage) by roughly 1.66%. This advancement directly improves the energy efficiency of serverless systems at the network edge.

**Table 3:** A comparative study on energy waste (optimized consumption), failure resistance, and uptime across scheduling methods under diverse workload types.

Workload types	Energy loss proportion by strategy			
	*IAOA*	*HBI*	*BBO*	Proposed
Exponential	${E}_{IAOA}$ = 1.67% (486 mWh)	${E}_{HBI}$ = 1.99% (530 mWh)	${E}_{BBO}$ = 1.33% (430 mWh)	${E}_{proposed}$ = 0%
Poisson	${E}_{IAOA}$ = 1.71% (490 mWh)	${E}_{HBI}$ = 1.81% (508 mWh)	${E}_{BBO}$ = 1.63% (481 mWh)	${E}_{proposed}$ = 0%
Exponential-poisson	${E}_{IAOA}$ = 1.40% (440 mWh)	${E}_{HBI}$ = 1.59% (460 mWh)	${E}_{BBO}$ = 1.79% (500 mWh)	${E}_{proposed}$ = 0%

Position levels	Resistance to failure of approaches during set break rounds			
	*IAOA*	*HBI*	*BBO*	Proposed
Position (1)	${R}_{IAOA}$ = 16 (Break)	${R}_{HBI}$ = 22 (Break)	${R}_{BBO}$ = 28 (Break)	${R}_{Proposed}$ = 16 (Continue) ${R}_{Proposed}$ = 22 (Continue) ${R}_{Proposed}$ = 28(Continue)
Position (2)	${R}_{IAOA}$ = 16 (Break)	${R}_{HBI}$ = 26 (Break)	${R}_{BBO}$ = 26 (Break)	${R}_{Proposed}$ = 16 (Continue) ${R}_{Proposed}$ = 26 (Continue)
Position (3)	${R}_{IAOA}$ = 14 (Break)	${R}_{HBI}$ = 14 (Break)	${R}_{BBO}$ = 14 (Break)	${R}_{Proposed}$ = 14 (Continue)

Workload types	Uptime percentage of approaches			
	*IAOA*	*HBI*	*BBO*	Proposed
Exponential	${Up}_{IAOA}$ = 91.9% (Up ${R}_{IAOA}\le 32\text{ R}$)	${Up}_{HBI}$ = 94.6% ($\text{Up }{R}_{HBI}\le 31\text{ R}$)	${Up}_{BBO}$ = 91.9% (Up ${R}_{BBO}\le 32\text{ R}$)	${Up}_{proposed}$ = 100% (35 Up ${R}_{Proposed}\le +\text{R}$)
Poisson	${Up}_{IAOA}$ = 91.9% (Up ${R}_{IAOA}\le 32\text{ R}$)	${Up}_{HBI}$ = 91.9% ($\text{Up }{R}_{HBI}\le 32$)	${Up}_{BBO}$ = 91.9% ($\text{Up }{R}_{BBO}\le 32\text{ R}$)	${Up}_{proposed}$ = 100% (35 Up ${R}_{Proposed}\le +\text{R}$)
Exponential-poisson	${Up}_{IAOA}$ = 91.9% ($\text{UP }{R}_{IAOA}\le 32\text{ R}$)	${Up}_{HBI}$ = 89.2% (Up ${R}_{HBI}\le 31$)	${Up}_{BBO}$ = 89.2% ($\text{Up }{R}_{BBO}\le 31\text{ R}$)	${Up}_{proposed}$ = 100% (35 Up ${R}_{Proposed}\le +\text{R}$)

#### Situation 2: Evaluation of failure resistance

**Fig. 9:**
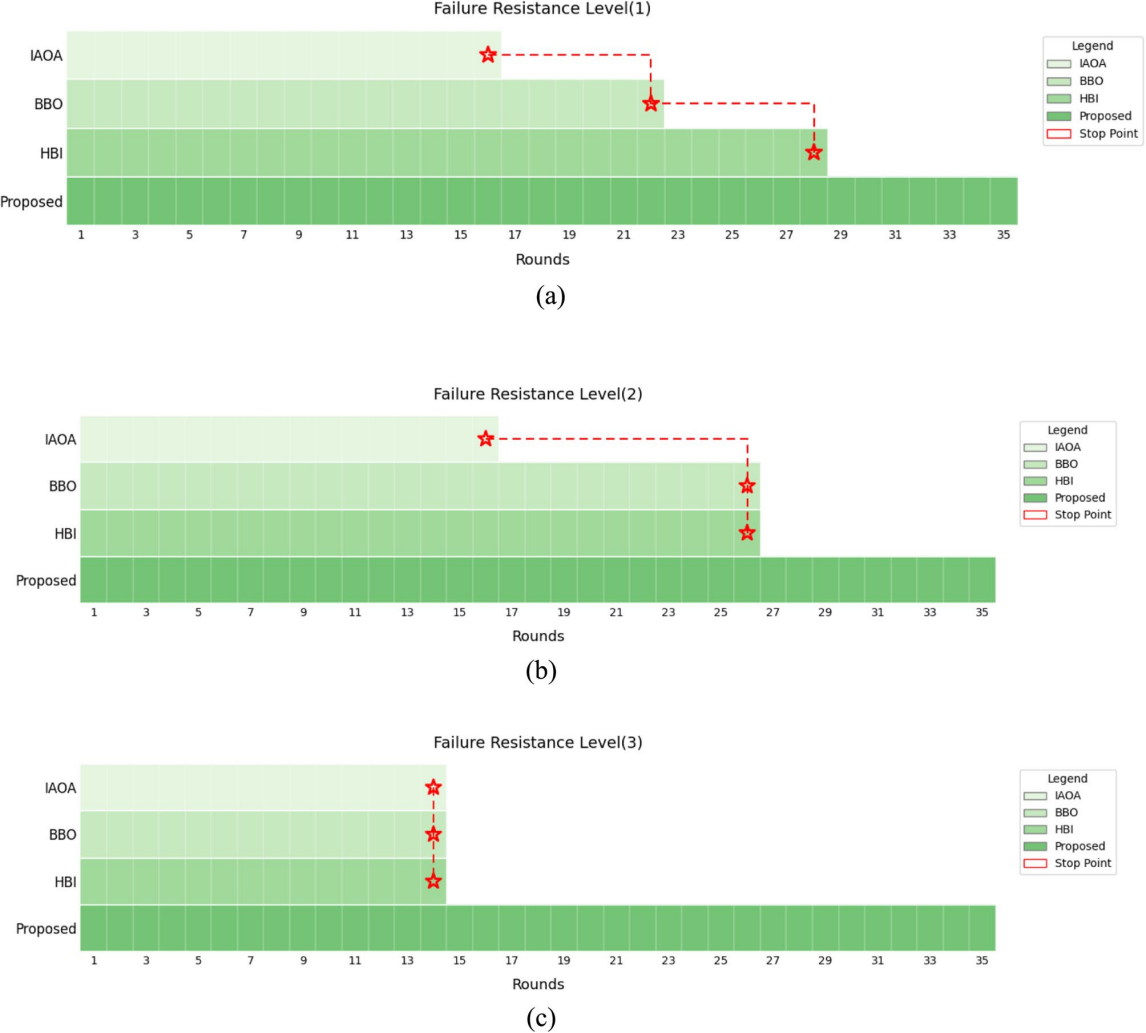
Evaluation of network availability under failure resistance conditions.

Table 3 shows that the proposed approach delivers outstanding failure resistance performance during scheduler breaks. At the position (1), although IAOA (${R}_{IAOA}$ = 16 Break), HBI (${R}_{HBI}$ = 28 Break), and BBO (${R}_{BBO}$ = 22 Break) (Fig. 9a) suffered interruptions, the proposed approach employed its self-healing MAPE‑based cycle to keep operating in rounds ${R}_{proposed}$ = 16, ${R}_{proposed}$ = 28 and ${R}_{proposed}$ = 22 break. At the position (2), although IAOA (${R}_{IAOA}$ = 16 Break), HBI (${R}_{HBI}$ = 26 Break), and BBO (${R}_{BBO}$ = 26 Break) (Fig. 9b) suffered interruptions, the proposed approach employed its self-healing MAPE‑based cycle to keep operating in rounds ${R}_{proposed}$ = 16, and ${R}_{proposed}$ = 26 break .At the position (3), although IAOA (${R}_{IAOA}$=14 Break), HBI (${R}_{HBI}$ = 14 Break), and BBO (${R}_{BBO}$ = 14 Break) suffered interruptions, the proposed approach employed its self-healing MAPE‑based cycle to keep operating in rounds ${R}_{proposed}$ = 14 break (Fig. 9c), whenever an existing scheduler was interrupted, the mechanism instantly switched to an alternative scheduler, preventing system unavailability. Ultimately, it achieved a 100% performance improvement over competing approaches. Moreover, combining offloading functions with APU resources during energy crises, the proposed framework design boosts energy efficiency and guarantees system stability and availability under potential failure resistance conditions.

#### Situation 3: Measuring network uptime duration

**Fig. 10:**
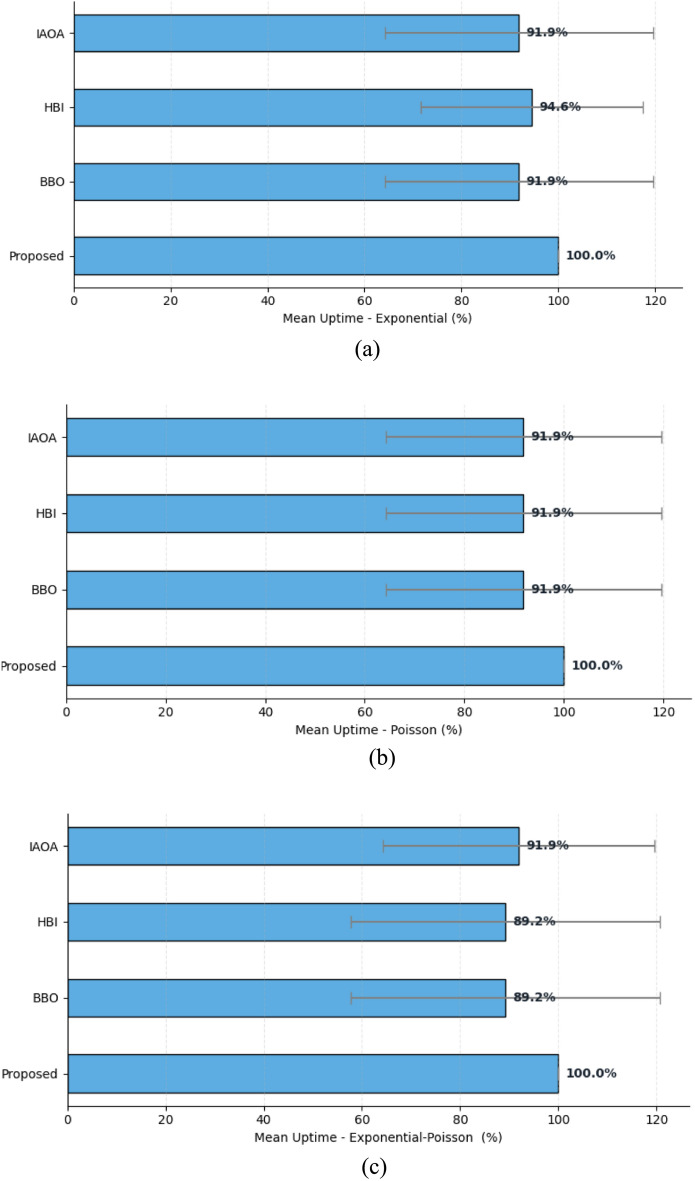
Mean uptime performance of scheduling approaches under Exponential (**a**), Poisson (**b**), and Exponential-poisson (**c**) Workloads.

The experimental results in Table 3 confirm that our proposed Uptime mechanism for serverless systems at the network edge consistently outperforms the IAOA, HBI, and BBO approaches. Hence, under the Exponential, Poisson, and Exponential-Poisson workloads are respectively equal to IAOA (${Up}_{IAOA}$ = 91.9%), HBI (${Up}_{HBI}$ = 94.6%, 91.9%, and 89.2%) Uptime and BBO (${Up}_{BBO}$ = 91.9% and 89.2%) with Active ${R}_{IAOA,HBI,BBO}$ ≤ 32 or 31 constraints. In contrast, our method sustains 100% Uptime (up to 35 Active ${R}_{proposed}$ ≤  + $R$) by offloading functions to the APU as soon as device energy drops to the critical 10% level. The same superior behavior appears in the Exponential (Fig. 10a), Poisson (Fig. 10b), and Exponential‑Poisson (Fig. 10c) patterns, where traditional algorithms deliver just 89.2 to 94.6% Uptime, yet our energy level awareness approach delivers defect‑free 100% Uptime. This strategy boosts overall system Uptime by roughly 8.6%, enhancing access latency and stability for edge‑deployed serverless applications.

### Considerations and limitations of the proposed method

While the automatic energy-conscious scheduling framework performs well in optimizing energy consumption and increasing accessibility in edge environments, it may need to optimize monitoring processes and analysis to maintain large-scale efficiency in large networks with high nodes. It also requires more advanced mechanisms of energy estimation in low-income communication or highly heterogeneous communication, precise timing matching with the actual status of the nodes. These are not the inherent limitations of the framework, but rather an opportunity for future development.

### Comparison with deep reinforcement learning algorithms

Deep Reinforcement Learning (DRL) algorithms (by the ability to learn optimal decision-making policies in dynamic and complex environments) are considered one of the potential options for energy-aware scheduling management in edge networks. However, our proposed method based on the MAPE framework and energy-aware prescheduling has some advantages over DRL algorithms. These advantages include reduced computational overhead, no need for a long training period and large data sets, fast and real-time response to energy changes, and ease of deployment in resource-constrained nodes. In contrast, DRL algorithms usually require abundant training data, long convergence time, and high processing power to learn optimal policies, which can be challenging in real IoT environments with limited energy and processing resources.

## Conclusion

IoT devices deployed at the network’s edge face significant resource constraints, such as limited processing power, low memory, and high-power consumption. These challenges can reduce system performance, increase latency, and reduce battery life. To overcome these obstacles, combining the serverless computing paradigm with an energy-aware scheduling approach can improve the performance of edge devices by intelligently allocating resources and optimizing energy consumption. This paper introduces an AEAS based on the MAPE-based control loop. In the pre-scheduling phase, the energy levels of active nodes in the network are monitored, and based on the predicted energy state, an appropriate scheduler is selected to process requests. This dynamic decision-making enables resource allocation at runtime to be tailored to energy fluctuations and avoid unnecessary energy waste. In addition, the proposed mechanism also includes the use of failure resistance solutions and increased availability of active nodes so that the uptime duration is maximized in the face of energy reduction. The efficiency of this method was evaluated in three different load distribution patterns exponential, poisson, and exponential-poisson. The results show that an average of 1.66% reduces energy consumption and improves network uptime by 8.6% compared to other methods. The proposed mechanism provides stable and uninterrupted performance and failure resistance guarantees in all scenarios. These achievements confirm the high effectiveness of the introduced approach in optimizing energy consumption and increasing the resilience and stability of serverless computing-based systems.

In future work, we plan to improve the AEAS by adding reinforcement learning capabilities. Specifically, the scheduler will bootstrap its availability by relying on data collected from past choices and using reinforcement learning algorithms to learn optimal resource allocation policies when the system cannot reach the monitoring phase.

## Data Availability

The datasets used or analyzed during the current study are available from the corresponding author on reasonable request.
